# Role of Hypoxia and Metabolism in the Development of Neointimal Hyperplasia in Arteriovenous Fistulas

**DOI:** 10.3390/ijms20215387

**Published:** 2019-10-29

**Authors:** Nirvana Sadaghianloo, Julie Contenti, Alan Dardik, Nathalie M. Mazure

**Affiliations:** 1 Centre de Méditerranéen de Médecine Moléculaire (C3M), Université Côte d’Azur, INSERM U1065, 151 Route de St Antoine de Ginestière, BP2 3194, 06204 Nice CEDEX 03, France; contenti.j@chu-nice.fr (J.C.); mazure@unice.fr (N.M.M.); 2 Department of Vascular Surgery, Centre Hospitalier Universitaire de Nice, 06000 Nice, France; 3 Department of Emergency Medicine, Centre Hospitalier Universitaire de Nice, 06000 Nice, France; 4 Department of Surgery and the Vascular Biology and Therapeutics Program, Yale University, New Haven, CT 06520, USA; alan.dardik@yale.edu; 5 Department of Surgery, VA Connecticut Healthcare Systems, West Haven, CT 06516, USA

**Keywords:** arteriovenous fistula, hypoxia, hypoxia-inducible factor, metabolism, neointimal hyperplasia

## Abstract

For patients with end-stage renal disease requiring hemodialysis, their vascular access is both their lifeline and their Achilles heel. Despite being recommended as primary vascular access, the arteriovenous fistula (AVF) shows sub-optimal results, with about 50% of patients needing a revision during the year following creation. After the AVF is created, the venous wall must adapt to new environment. While hemodynamic changes are responsible for the adaptation of the extracellular matrix and activation of the endothelium, surgical dissection and mobilization of the vein disrupt the *vasa vasorum*, causing wall ischemia and oxidative stress. As a consequence, migration and proliferation of vascular cells participate in venous wall thickening by a mechanism of neointimal hyperplasia (NH). When aggressive, NH causes stenosis and AVF dysfunction. In this review we show how hypoxia, metabolism, and flow parameters are intricate mechanisms responsible for the development of NH and stenosis during AVF maturation.

## 1. End-Stage Renal Disease and Arteriovenous Fistulas

### 1.1. Chronic Kidney Disease

Chronic kidney disease (CKD) describes the gradual loss of kidney function. At the stage of kidney failure, also called end-stage renal disease (ESRD) renal function must be replaced by either a new functioning kidney or an artificial kidney in order to maintain life. The main etiology of CKD is related to the diabetes-metabolic syndrome-obesity bundle and hypertension. As the prevalence of these diseases is growing exponentially worldwide, so is CKD [1]. In 2016, 124,675 new cases of ESRD were reported in the USA, with a total of nearly 500,000 patients receiving dialysis treatment and over 200,000 patients living with a kidney transplant [2]. Hemodialysis is the most commonly utilized therapeutic approach for the treatment of ESRD in the majority of countries [3]. Current hemodialysis devices run at a flow of 300 to 400 mL/min, meaning that the blood flow should be at least 500 to 600 mL/min [4]. As a comparison, the standard blood flow rate in a forearm radial artery is less than 40 mL/min [5]. Several types of vascular access have been designed and used for that purpose.

### 1.2. Arteriovenous fistulas (AVFs)

**Figure 1:**
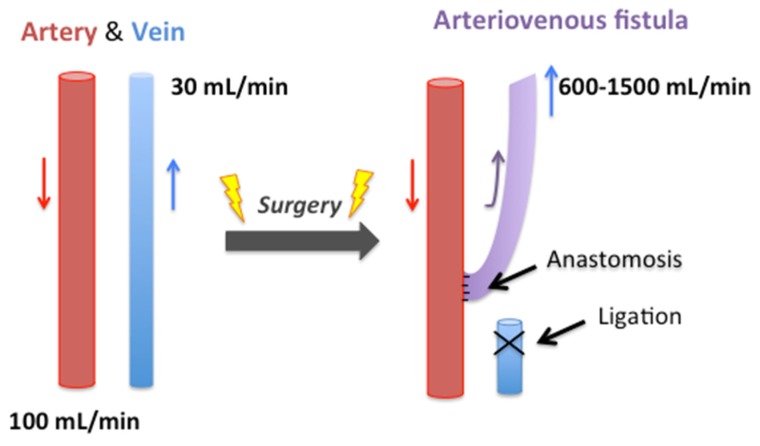
A fistula is direct communication between a vein and an artery, frequently on the upper limb of the patient. This communication is made by the surgeon with the help of a suture. The vein is dissected and ligated its extremity, and sewn to the artery. The arterial blood (red arrow) flows into the vein (blue arrow) through this anastomosis. The fistula matures, and the blood flow in the fistula (purple arrow) reaches around 1 L per minute.

Hemodialysis arteriovenous fistulas (AVFs) are direct communications between a vein and an artery of the patient (Figure 1), first described in 1966, by Brescia et al. [6]. Bringing the arterial flow to the vein induces a large increase in the venous flow. With progressive maturation, which usually takes around six weeks, the venous wall becomes thicker, the venous diameter larger (>5–6 mm), and the venous flow reaches 600 to 1200 mL/min [5]. 

AVFs are considered the optimal vascular access for hemodialysis because of their potential for durability, lower risk of infection, and reduced need for intervention to ensure patency compared to grafts or catheters; however, literature and registries reveal a high prevalence of failure of newly placed fistulas never becoming functional (early failure) [4,7]. Based on the Unites States Renal Data System (USRDS), among AVFs placed between June 2014 and May 2016, 39% failed to mature sufficiently for use in dialysis. Of those that matured and were finally used, the median time to the first use was 108 days [2]. Late failure worsen the outcome of these AVFs, with a recent systematic review and meta-analysis reporting that primary patency rates of AVFs were 60% at 1 year and 51% at 2 years, meaning almost half of the patients had at least one reintervention on their AVF to maintain patency [8]. Secondary patency rates of 71% at 1 year and 64% at 2 years, meaning 30–40% of all AVFs were abandoned after a short time [8]. These reinterventions add to the burden of hemodialysis patients, already tired by the disease, the 3-times a week visit to the dialysis facility, the dialysis sessions themselves, and all the burden of vascular access monitoring [9]. Vascular access care is also responsible for a significant proportion of health care costs, in particular in the first year of hemodialysis. In the USRDS report, the cost of hemodialysis treatment was evaluated in the US at $90,971 per patient per year [2].

These data show that even the AVF, the best vascular access for hemodialysis, is clearly sub-optimal for a permanent treatment. The vascular access and dialysis community needs to better understand and correct the mechanisms leading to AVF failure.

## 2. Pathophysiology of AVFs

### 2.1. How AVFs Mature

During AVF creation, the surgeon directly connects the artery, a high-pressure pulsatile flow conduit, to a vein, which is ordinarily a low-pressure steady flow conduit (Figure 1). The pressure gradient results in an immediate increase in flow in both the artery and vein. The resulting hemodynamic change initiates a vascular remodeling response within both vessels. Pressure in the venous segment increases upon AVF creation and remains relatively constant during the time course of remodeling and thereafter. Outward remodeling is thought to be mediated by the venous endothelium and adventitia that sense these hemodynamic forces and integrates them to allow successful adaptation without loss of luminal area and vessel patency [10]. Venous diameter expansion is a critical element of outward remodeling and predicts the clinical success of AVF [11].

**Figure 2:**
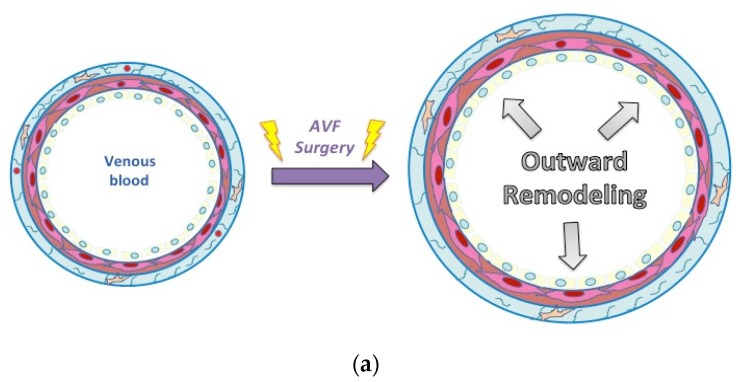

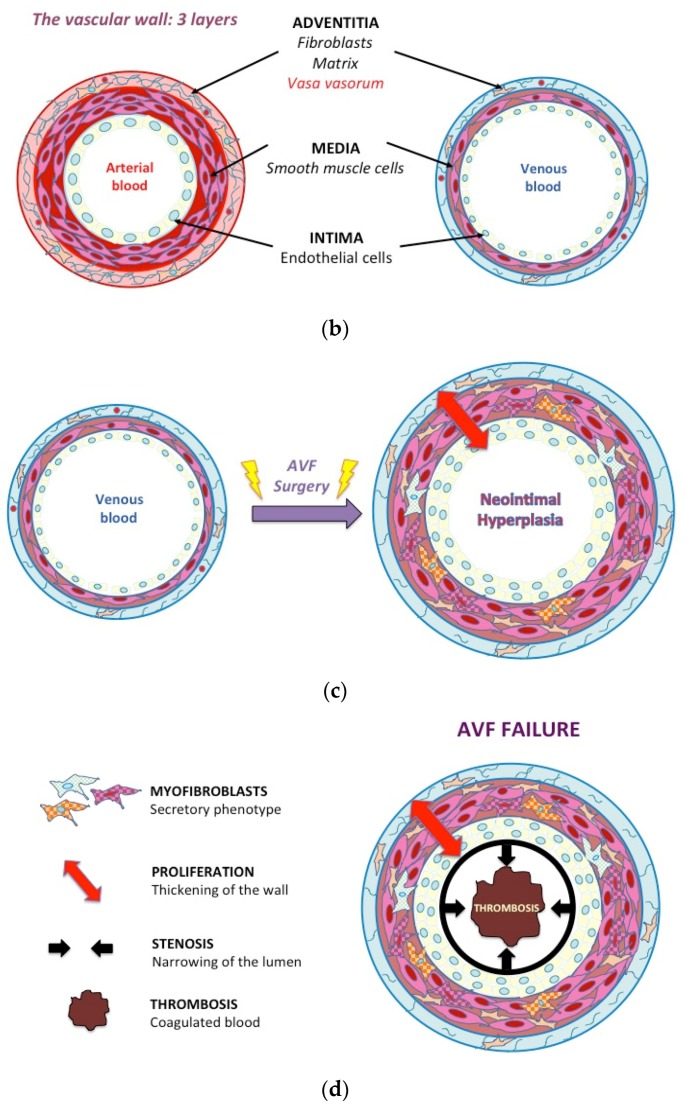
Mechanisms of venous wall remodeling. (**a**) Similar to the arterial wall, the venous wall is composed of three layers: the external layer, called the adventitia, composed of extracellular matrix (ECM), fibroblasts, immune cells, and *vasa vasorum*. The middle layer, called the media, is made of smooth muscle cells (SMC) and some extracellular fibers. The internal layer, also called intima, is composed of endothelial cells. (**b**) and (**c**), upon arteriovenous fistula (AVF) creation, in response to the altered hemodynamic environment, several structural changes occur in the vessel wall. Surgery induces endothelial denudation, matrix reorganization to accommodate outward expansion but also vascular SMC proliferation and migration, contributing to NH formation. (**d**) Aggressive intimal hyperplasia induces stenosis (narrowing) of the vessel that can provoke thrombosis (occlusion).

The venous wall is composed of three layers (Figure 2A) [12]. The external layer, called the adventitia, acts like a thin but resistant envelope to the vessel. It is composed of extracellular matrix (ECM), fibroblasts and immune cells. *Vasa vasorum*, small vessels, also run around the vein within the adventitia, to bring arterial blood flow to the venous wall [13]. The middle layer, called the media, is made of smooth muscle cells (SMC) and some extracellular fibers. The internal layer, also called intima, is composed of endothelial cells. During remodeling, hemodynamic changes and surgical injuries have an impact on all three layers of the venous wall. Endothelial and adventitial signaling induces, in turn, structural changes in the cells and the ECM, which affect the venous caliber and thickness (Figure 2B,C). Lack of outward remodeling and/or excess in wall thickening creates a disequilibrium that leads to AVF non-maturation and early failure (Figure 2D) [12].

### 2.2. Flow and Hemodynamic Parameters in AVF

The increased hemodynamics of arterial flow that increases vessel wall shear stress (WSS) are critical events after AVF creation that promotes AVF adaptation [14]. In a patient-specific end-to-side fistula, image-based computational fluid dynamics studies showed laminar flow within the arterial limb and a complex multi-directional and reciprocating flow field on the inner side of the swing segment in the proximal venous limb [15]. Wall thickening is predisposed to occur in the inner wall of the venous segment near the anastomosis and has a strong inverse correlation with magnitudes of shear stress, but is also related to flow patterns [16]. Although data is sparse in the venous vasculature and venous cells, studies on the arterial vasculature have shown that disturbed flow, with low and reciprocating WSS, is known to induce selective expression of atherogenic and thrombogenic genes that are pro-oxidant, proinflammatory, procoagulant, and proapoptotic in endothelial cells [17,18], and to stimulate vascular smooth muscle cell migration and proliferation, all of which may enhance wall thickening [19,20,21]. 

## 3. Neointimal Hyperplasia in AVF

### 3.1. Definitions

Wall thickening is the adaptation of the vessel wall to increased pressure and abnormal WSS. This process involves the expansion of all the vessel layers via ECM deposition, cell proliferation and migration [22]. The thickening of the venous wall composed of these matrix proteins and proliferative and secretive cells is called neointimal hyperplasia (NH). Excessive neointimal hyperplasia causes stenosis. As a consequence of stenosis, the blood flow may be diminished and the AVF becomes unusable for dialysis. In extreme cases, stenosis may cause thrombosis, a complete occlusion of the vessel because of non-circulating blood (Figure 2D). Several types of cells are involved in NH, including ECs, SMCs, and adventitial fibroblasts [23].

### 3.2. Role of Endothelial Cells

The release of chemotactic and inflammatory mediators from the endothelium during surgical manipulation and hemodynamic variation are substantial during the initial phase of adaptation [24]. Directly after AVF creation, high magnitudes of arterial flow result in both passive vascular distention and nitric oxide (NO) synthesis by endothelial cells with subsequent vascular SMC relaxation, resulting in acute vasodilation [25,26]. NO is produced by endothelial nitric oxide synthase (eNOS) and may contribute to adaptive vein wall remodeling through its anti-inflammatory, anti-thrombotic, and antiproliferative properties. Both eNOS and inducible nitric oxide synthase (iNOS) are upregulated in the AVF, and pharmacological inhibition of eNOS results in increased monocyte chemoattractant protein-1 and interleukin (IL) 8, leading to NH [27]. Endothelin-1 (ET-1) is an inflammatory mediator of vasoconstriction and endothelial proliferation. ET-1 expression is upregulated in the venous wall and within areas of NH in AVF as well as in the plasma of patients with chronic renal failure and hemodialysis. ET-1 may mediate wall thickening in response to localized hemodynamic forces [28,29]. Selectins facilitate leukocyte adhesion. P-selectin is present on ECs and platelets, and E-selectin is present on ECs. Intercellular adhesion molecule (ICAM) and vascular cell adhesion molecule (VCAM) facilitate additional binding and migration [30]. P-selectin and E-selectin expression are both upregulated early after AVF creation, followed by decreased P-selectin expression after 1 month [27]. VCAM-1, but not ICAM-1, is highly expressed in thrombosed and stenotic AVF [31].

### 3.3. Role of Smooth Muscle Cells

The canonical origin of proliferating cells in NH is the vessel media that contains SMC. Endothelial and smooth muscle cell injury, consequences of hemodynamic stress and mechanical injury, results in the migration of SMCs from the media into the intima, where the SMC proliferate and differentiate into a secretory phenotype (myofibroblasts). This process of injury, followed by migration, proliferation and differentiation, is orchestrated by a large number of mediators, such as cell cycle regulators (p27-p16 [32], p38 mitogen-actived protein kinase [33]) and growth factors (vascular endothelial growth factor (VEGF) [34], platelet derived growth factor (PDGF) [27], basic fibroblast growth factor (bFGF) [35,36], insulin-like growth factor-1 (IGF-1) [37], etc.). Expression of adhesion molecule β-catenin and proto-oncogene c-Myc is increased one week after AVF creation, correlating with decreased adhesion molecule N-cadherin, associated with vascular SMC proliferation [38]. Vascular SMC may also become resistant to NO, decreasing vascular SMC relaxation and preventing AVF maturation by reducing outward remodeling [39]. As shown recently, mature SMCs are keys to the equilibrium between medial wall thickening (proliferation of differentiated SMCs that promotes maturation) and neointimal hyperplasia (dedifferentiated SMCs that cause NH and failure) [40]. Interestingly, vascular SMCs from the proximal artery may also contribute to venous NH: in a murine AVF model, increased Notch signaling could drive migration of these cells to the venous outflow tract [41].

### 3.4. Role of Fibroblasts

More recent studies have shown that the adventitial fibroblasts are critical during venous adaptation to arterial flow and help maintain venous wall integrity and hemostasis after surgical creation of the AVF [23,42]. Fibroblast precursors residing in the venous adventitia sense the abrupt changes in mechanical forces produced by the arterial flow to rapidly adjust their genomic expression program to help increase vascular resistance [43]. They differentiate into myofibroblasts, form matrix bundles of contractile microfilaments and extensive cell-to-matrix attachment sites, and secrete matrix metalloproteinases (MMPs), collagen, and ECM proteins that strengthen the fistula wall. Recent data suggest that NH in AVFs consists mainly of smooth muscle α-actin-positive, vimentin-positive and desmin-negative myofibroblasts that have probably migrated from the adventitial layer [23,44]. Analysis of venous segments of failed AVF has shown increased adventitial fibrosis, myofibroblast activation, and capillary rarefaction [45].

### 3.5. Role of Inflammatory Cells

Inflammatory cells and cytokines, such as CD68-positive macrophages, CD3-positive lymphocytes, IL-6, IL-8, and monocyte chemo-attractant protein-1 are also known to be part of the NH process in AVFs [29,46,47,48]. This inflammatory state, exacerbated by CKD and dialysis, leads towards the up-regulation of numerous growth factors and cytokines, such as insulin-like growth factor-1, platelet-derived growth factor and basic fibroblast growth factor [27,37]. They all play a significant role in the stimulation of cell proliferation and migration. Even transforming growth factor-β1, usually known for its anti-inflammatory properties, is produced by ECs, vascular SMCs and inflammatory cells within the venous wall, leading to ECM deposition. These cells also participate in NH in AVFs [36,37,49].

### 3.6. Role of Extracellular Matrix

The equilibrium between ECM degradation and accumulation is closely related to the maturation process of AVFs. In a murine AVF model, Hall et al. showed that initial ECM degradation occurs early after AVF creation, coincident with an early increase in expression of matrix metalloproteinases (MMP-2 and -9) and tissue inhibitor of metalloproteinase-1 (TIMP-1) [50]. Although the role of TIMP-1 is not clear, an imbalance in MMP and TIMP may contribute to AVF failure [51]. By day 7, there is an increase in collagen and elastin production and a change in patterns of MMP expression. By day 21, expression of MMPs is reduced and expression of larger structural and non-collagenous matrix proteins, such as fibronectin and perlecan, is increased. Other studies have shown that decreased expression of MMP-1, MMP-3, and MMP-9 is linked to increased AVF failure and stenosis [52]. Giachelli et al. have found that the expression of osteopontin (OPN) was up-regulated in the development of NH in a model of arterial balloon-injury. OPN was originally identified as matrix protein in bones, facilitating cell adhesion and migration, but this secreted protein has also been found in several experimental models of AVF maturation [50,53]. Its pro-inflammatory properties may favor vascular remodeling and development of NH [54].

### 3.7. Dissection Injury: Ischemia and Oxidative Stress 

**Figure 3:**
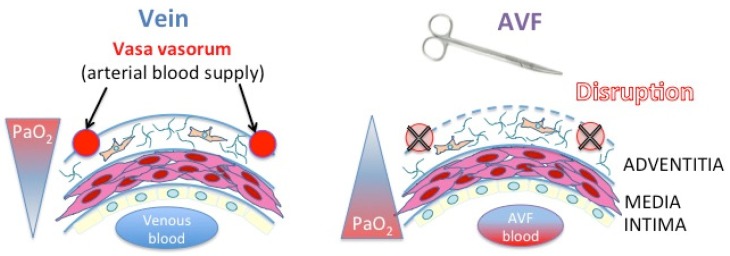
Changes in the venous wall after surgery: The *vasa vasororum* disruption creates a new oxygen gradient. The *vasa vasorum* in the adventitia provides oxygenated blood to the wall, whereas the intima is in contact with a venous blood low in oxygen. During surgery, the vein is dissected, disrupting *the vasa vasorum*, and potentially decreasing oxygen levels. On the other side, oxygenated arterial blood mixed to venous blood potentially creates an inversion of the oxygen gradient.

Hemodynamic changes and surgery-induced mechanical injury to the venous wall have been proposed as the etiology of juxta-anastomotic stenosis and maturation failure of AVF; however, a more recent theory proposes that hypoxia and oxidative stress may also play a role. Starting from the observation that during traditional AVF creation the vein is dissected free from the surrounding tissue, and that this same dissected segment is responsible for the majority of stenosis, researchers hypothesized that the rupture of the vasa vasorum during surgical dissection may induce hypoxia, low oxygen (O_2_) concentration, in the venous wall [12,34] (Figure 3). Additionally, the O_2_ partial pressure (pO_2_) in the venous blood is approximately 35–45 mmHg. However, after AVF creation, pO_2_ in the fistula increases to the level of arterial pO_2_ (73–100 mmHg). Thus it is possible that interruption of the arterial blood supply followed by the sudden increase in luminal pO_2_ causes an ischemia-reperfusion phenomenon within the venous wall. Both of these phenomena provoke oxidative stress and stimulate numerous signaling pathways [36]. Interestingly, Joddar et al. have shown that arterial levels of oxygen stimulate NH in veins via a ROS-dependent mechanism [55]. In addition, two computational studies suggest that hypoxic segments of vessels may be linked to hemodynamic patterns and may correlate with stenosis [56,57]. Thus the two pathophysiological theories, flow and ischemia, may be related.

## 4. The Hypoxic Pathway in AVF 

### 4.1. Hypoxia-Inducible Factors (HIFs), Keys to the Hypoxic Environment

Maintaining oxygen homeostasis is essential for the survival of all aerobic organisms. Therefore, a change in pO_2_ is a major physiological stimulus that elicits rapid cellular responses. HIFs are key transcription factors that regulate the cellular response to hypoxic injury by stimulating the transcription of genes that help restore O_2_ and maintain effective energy production [58]. HIFs belong to the large family of basic-helix-loop-helix transcription factors and bind DNA as a heterodimer composed of one oxygen-regulated α-subunit (isoforms HIF-1α, HIF-2α, or HIF-3α) and two β stable subunits (HIF-1β or aryl hydrocarbon receptor nuclear translocator (ARNT), and HIF-2β or ARNT2) [59,60].

### 4.2. Oxygen-Dependent Regulation of HIFs

**Figure 4:**
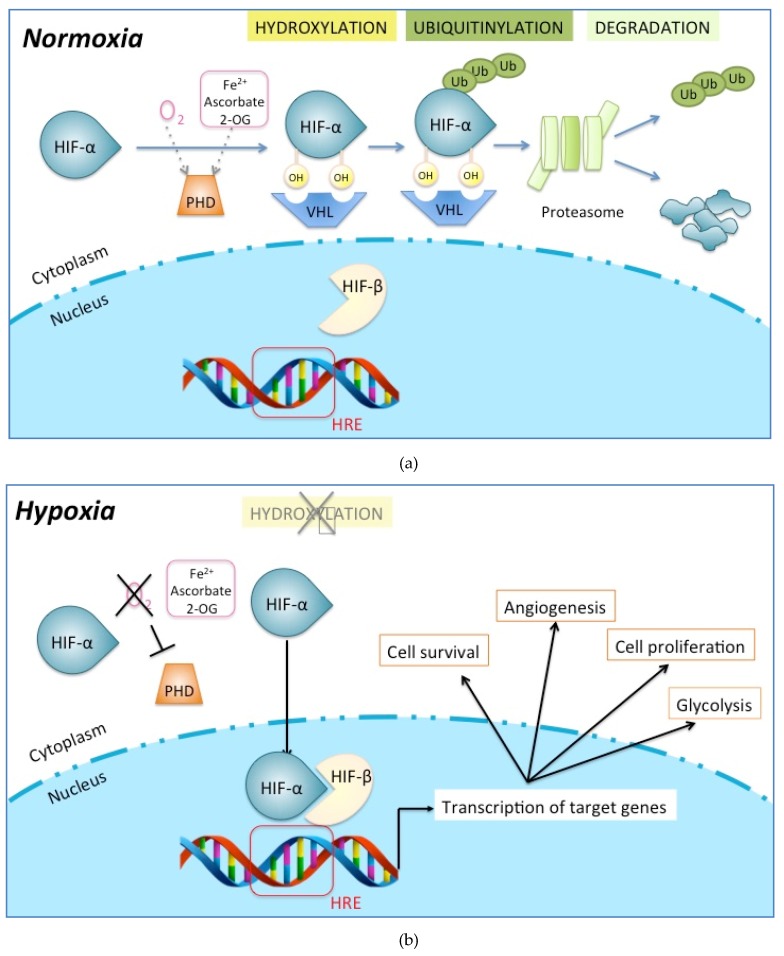
Molecular mechanisms of oxygen sensing and signaling. (**a**) In normoxia, hypoxia-inducible factor-α subunit (HIF-α) is hydroxylated through a combination of α-ketoglutarate, molecular oxygen (O_2_), one or more of the prolyl hydroxylase (PHD) isoenzymes and the asparaginyl hydroxylase factor inhibiting HIF (grey dotted arrow). The von Hippel–Lindau protein (VHL)-containing E3 ubiquitin ligase targets the α-subunit for polyubiquitylation and degradation in the proteasome. (**b**) In hypoxia, low oxygen (black cross line) causes inactivation of PHD (black T arrow). The α-subunit is no longer hydroxylated (grey cross line) and becomes stabilized. In the nucleus, HIF-α binds to the HIF-β subunit and with the help of cofactors, the complex becomes transcriptionally active upon binding to the hypoxia-responsive element (HRE, red box) consensus sequence on the promoter of target genes. HIF-target genes are numerous, with key roles in cell survival, angiogenesis, proliferation, and glycolysis.

The discovery of molecular mechanisms behind oxygen-dependent regulation of HIFs was rewarded in 2019 by the Nobel Prize in Physiology or Medicine. In well-oxygenated cells, the HIF-α subunits are constantly and rapidly degraded by hydroxylation of proline residues within the oxygen-dependent degradation domain of HIF-α, with one of the shortest half-lives (less than 5 min) among cellular proteins (Figure 4A) [59,61,62]. Prolyl hydroxylation targets the HIF-α proteins for proteasomal degradation by promoting their interaction with the protein von Hippel-Lindau (pVHL) (Figure 4A) [59,61,63,64,65]. This post-translational modification of HIF-α is carried out by a family of prolyl hydroxylase enzymes, the prolyl hydroxylase domain proteins (PHD1, PHD2, PHD3) (Figure 4A) [61,62,66]. Since these enzymes bind oxygen directly, they are critical oxygen sensors within the hypoxic response pathway [67]. In hypoxia, these enzymes being inactivated, HIF-α proteins are not degraded and accumulate in cells (Figure 4B) [68]. An additional hydroxylation of an asparaginyl moiety at the end of the COOH-terminal transactivation domain of HIF-1α or HIF-2α subunit is catalyzed by an enzyme called factor-inhibiting HIF-1 (FIH-1) [69,70]. In normoxia, FIH-1 abrogates HIF transcriptional activity by inhibiting the binding of coactivators such as p300 and C-AMP Response Element-binding protein [71,72,73]. Under hypoxia, PHD inactivation causes HIF-α accumulation, and FIH-1 inactivation allows HIF transcriptional activity. While the HIF-β/ARNT subunits are constitutively expressed in the nucleus, the HIF-α subunits found in the cytoplasm have sensitivity towards O_2_. When the HIF-α subunit is stabilized and active, it leaves the cytoplasm to enter the nucleus, binds its β-subunit and cofactors, and induces the transcription of a large number of genes (1–2% of all human genes) (Figure 4B) [61]. All these genes have in common a hypoxia-responsive element (HRE: 5′ -RCGTG-3′) as an enhancer, either in their promoter, in the 3′ untranslated region, or in introns [58,59,60,61,62,63,64,65,66,67,68,69,70,71,72,73,74,75,76]. These genes have key biological roles such as cell survival (*ADM* [77], *cyclin D1* [78], *BNIP3* [79], *NIX* [80]), cell proliferation (*cyclin G2* [81], *IGF2* [82], *TGFα* [83], *NOS2* [84]), cell motility (*AMF/GPI* [83], *LRP1* [81]), cytoskeletal structure and ECM (*vimentin* [83], some keratins [83]), erythropoiesis (*EPO)* [85], pH regulation (*carbonic anhydrase 9 and 12*) [86], nucleotide metabolism (*adenylate kinase 4)* [87]), amino acid metabolism (*transglutaminase 2*) [81], iron metabolism (*transferrin* [88], *ceruleoplasmin* [89]), and glucose and energy metabolism (*HK2* [90], *GLUT1* [91], *GAPDH* [92], *LDHA* [93], *leptin* [94]). 

A number of HIF-target genes also control angiogenesis: (VEGF [95], VEGF receptor (VEGFR)-1 and -2 [96]), vascular remodeling (angiopoetin-2 and -4, TIE1, TIE2) [97] and vascular tone (HO1 [98], NOS2 [84], ET1 [81]). As such, HIF pathways have been of great scientific interest for more than two decades, in particular in the fields of development [99,100], cancer [101,102] and cardiovascular diseases [103]. 

### 4.3. Oxygen-Independent Regulation of HIFs

HIF-α can also be stabilized in normoxic conditions as a consequence of cell injury and oxidative stress. Several O_2_-independant mechanisms of HIF regulation have been identified such as receptor for activated C kinase 1 (RACK1) [104], carboxyl terminus of Hsc70-interaction protein (CHIP) and heat shock protein-70 (HSP70) [105], hypoxia-associated factor (HAF) [106], and Sirtuin-7 (SIRT7) [107]. They all mediate ubiquitination and proteasomal degradation. HIF-1α responds also to non-hypoxic stimuli [68], including hormones like insulin [108,109], growth factors such as IGF-1 [110] or PDGF [111], vasoactive peptides such as nitric-oxide (NO) [112,113] or angiotensin 2 [111,114] and cytokines such as tumor necrosis factor-α (TNF-α) [115]. It is assumed that these O_2_-independent stimuli contribute to an autocrine role in proliferation, survival or tissue repair, allowing early adaptation to variations in the cell environment. 

Since many of these stimuli induce reactive oxygen species (ROS) production as part of their signaling cascade, it has been hypothesized that ROS participate directly in HIF stabilization [116]. Incubation of cells with H_2_O_2_ leads to the stabilization of HIF-α proteins and activation of HIF target genes even when O_2_ levels are high [117,118]. Furthermore, exposing keratinocytes to ultra-violet B irradiation activates HIF-1α through generation of mitochondrial ROS [119]. Using junD-deficient cells that exhibit chronic oxidative stress, Gerald et al. discovered ROS-dependent iron-mediated regulation of PHD enzymes [120]. Since enhanced H_2_O_2_ levels promote the oxidation of Fe(II) to Fe(III) through the Fenton reaction, it reduces the cellular pool of Fe(II) and increases even more drastically the proportion of active PHD. PHD and FIH-1 not only directly sense O_2_ concentrations, as discussed above, but are also regulated by ROS (mostly H_2_O_2_) and metabolites through modulation of iron availability [120,121,122] and 2-OG accessibility [121], respectively. In addition to PHD inactivation, ROS has also been shown to act on HIF expression via activation of kinases [123]. ROS-induced HIF-α stabilization was reversed in various studies by iron supplementation, reducing agents or antioxidant compounds. Treating cells with anti-oxidant compounds, such as the free radical scavenger *N*-acetylcysteine [124,125,126], glutathione [126], or vitamins E and C [127,128], markedly attenuates HIF-α protein accumulation and expression of HIF target genes in various cell types, including hepatoma cells [124], vascular SMC [129], myocardial cells [127], gastric epithelial cells [125], renal tubuloepithelial cells [114], and macrophages [126]. Therefore, oxidative stress could trigger activation of HIF even in well-oxygenated conditions, thereby mimicking hypoxia signaling. 

### 4.4. HIF-1 vs. HIF-2

The expression of HIF-2α was first believed to be restricted to blood vessels and in particular to vascular endothelial cells [85,130,131]. Since then, the expression of HIF-2α has been found in several tissues, including hypoxic kidney, lung, colonic epithelia, hepatocytes, macrophages, muscle cells and astrocytes [132]. Some early studies showed that besides common target genes, each HIF-α isoform seems to have unique targets [133,134], and this transcriptional specificity may reside in the NH_2_-terminal transactivation domain, suggesting that HIF-α interactions with transcriptional co-factors are key to determine differential gene activation [135]. However, recent studies have shown that the mechanisms leading to selective gene transactivation are highly context specific. The individual HIFs have specific temporal and functional roles: HIF-1 drives the initial response to hypoxia (<24h) and HIF-2 drives the chronic response (>24 h) [136,137]. They can also exhibit antagonistic functions on the same final target. While HIF-1α deletion in macrophages reduced the expression of iNOS and consequent production of NO [138], activation of HIF-2α in the same cell type will reduce the pool of L-arginine (the source of NO) by inducing arginase 1 [139]. 

The selective expression of HIF-1α vs. HIF-2α is the result of a complex regulation process rather than the sole consequence of cell type or O_2_-dependant stabilization of HIF-α subunits. HIF-1α vs. HIF-2α expression can be regulated selectively at the level of transcription, translation, or protein stability [133]. Lin et al. have shown that chromatin remodeling via differential acetylation of core histones in the promoter of HIF-1α or -2α may regulate their transcription [140]. Another well-known regulator of HIF-1α gene transcription is nuclear factor-KB (NF-KB), but it has not been shown to control HIF-2α [141]. As above, the phosphatidylinositol-3-kinases/protein kinase B/mechanistic arget of rapamycin (PI3K/AKT/mTOR) pathway regulates HIFα gene translation. However, Toschi et al. have shown that, in the same renal clear cell carcinoma cell line, HIF-1α expression was both mTOR complex 1 and 2 (mTORC1, mTORC2)-dependent, whereas HIF-2α was only mTORC2 dependent [142]. As for the stability of HIF-α subunits, HAF binds and leads HIF-1α towards proteasomal degradation under normoxic and hypoxic conditions in a pVHL-independent manner, but does not decrease HIF-2α levels [106]. In contrast, HAF binds HIF2α at a distinct C-terminal region and promotes HIF-2α transcriptional activity: cells switch from a HIF-1α to a HIF-2α transcriptional program [59,137]. In addition, HSP70 and CHIP bind and degrade HIF-1α (but not HIF-2α) under conditions of prolonged hypoxia in cultured cells, whereas rapid reoxygenation destabilizes both HIF-1α and HIF-2α proteins via the PHD-pVHL-proteasome pathway (Figure 4) [59,105]. This is one mechanism by which HIF-2 is considered to be the effector of prolonged hypoxia, whereas HIF-1 drives the initial response to hypoxia [59]. Other mechanisms of HIF differential regulation include sirtuins (SIRT1–7), a family of redox-sensitive, NAD^+^-dependent deacetylases and/or adenosine diphosphate (ADP)-ribosyltransferases, which are known to regulate complex changes in gene expression, metabolism, and redox status in mammalian cells [143]. SIRT1 acts at a post-translational level: forming a complex with HIF-2α, SIRT1 deacetylates lysine residues in the N-TAD, which enhances HIF-2α transcriptional activity [59,144]. However, deacetylatation of lysine residues in HIF-1α results in the repression of the HIF-1α transcriptional activity [145]; these opposing effects of SIRT1 may result in a switch towards either HIF-1α or -2α transcriptional programs in response to a metabolic switch in hypoxic tumors. The authors propose a mechanism of positive feedback in which under hypoxia HIF-1α promotes glycolysis, reducing NAD^+^/NADH (nicotinamide adenine dinucleotide, oxidized and reduced forms, respectively) ratios and inhibiting SIRT1, thereby further increasing the HIF-1α activity and presumably decreasing the HIF-2α activity [145]. Given the disparate actions and differential regulation of HIF-1 and -2, targeting the hypoxic pathway needs to determine whether broad or selective inhibition is required, depending on the cell type and the expected effect.

## 5. HIF and Cell Metabolism

**Figure 5:**
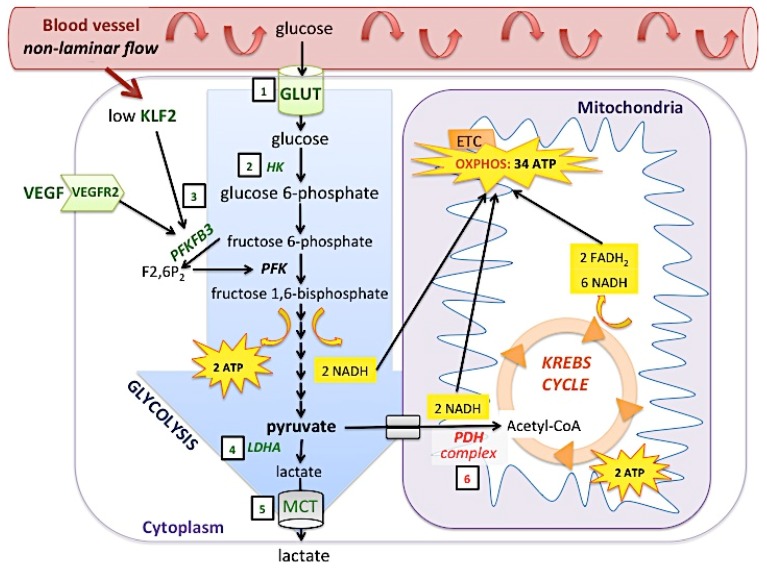
Glucose metabolism is regulated by hypoxia-inducible factors (HIFs). During glycolysis, glucose enters the cytoplasm through the glucose transporter (GLUT) and is transformed into glucose 6-phosphate by hexokinase (HK). Eight successive enzymatic reactions then occur to produce 2 molecules of pyruvate, 2 ATP and 2 NADH. At the end, cytosolic pyruvate has two possibilities: transformation into lactate by the enzyme lactate dehydrogenase A (LDHA)—lactate being then exported through the monocarboxylate transporter (MCT), or transferred into the mitochondria and transformed into Acetyl-CoA via the pyruvate dehydrogenase complex (PDH complex). In the second option, Acetyl-coA goes into the Krebs cycle to produce 2 ATP, 6 NADH, and 2 FADH_2_. NADH and FADH_2_ will further be used in redox reactions in the electron transport chain (ETC) to produce more ATP via oxidative phosphorylation (OXPHOS). HIF intervenes in several steps during glucose metabolism: by inducing GLUT it allows more entry of glucose (1) and by inducing hexokinase (2) and PFKFB3 (3), it enhances glycolysis. Note that non-laminar blood flow decreases the expression of Kruppel-like factor 2 (KLF2), which has otherwise an inhibitory action on HIF-1 and PFKFB3 expression. VEGF and its receptor VEGFR2 are also induced by HIF-1 and enhance PFKFB3 expression. Further, pyruvate is pushed towards lactate transformation by up-regulation of LDHA (4) and some isoforms of MCT (5). At the same time, HIF prevents the PDH complex from transforming pyruvate into Acetyl-coA (6). *Green: activation by HIF, Red: inhibition by HIF.*

Metabolism comprises a series of interconnected pathways that can function in the presence or absence of O_2_. Two of them, glycolysis and oxidative phosphorylation (OXPHOS) are represented in Figure 5. During glycolytic metabolism 1 molecule of glucose is converted into 2 molecules of pyruvate and generates 2 molecules of ATP and NADH. Lactate dehydrogenase A (LDHA) further reduces pyruvate into lactate [146]. During OXPHOS, 1 molecule of glucose is converted into 2 molecules of pyruvate, which are transported into the mitochondria, converted to acetyl coenzyme A and oxidized to CO_2_ in the Krebs cycle. The NADH and FADH_2_ generated in this process provide electrons to the electron transport chain in the inner mitochondrial membrane (respiratory cytochromes) [147]. Electrons are added to O_2_ at complex IV (cytochrome c oxidase or COX), finally generating 38 molecules of ATP per molecule of glucose (Figure 5).

### 5.1. Metabolic Adaptation to Hypoxia

Metabolic adaptation to hypoxia involves mainly a switch from oxidative (aerobic) to glycolytic (anaerobic) metabolism by cells. With this energy compensation, the cell continues to generate ATP and, despite the hypoxia, try to meet the metabolic demands and maintain the expenditure of energy of normoxic conditions. During hypoxia, HIF-1 stimulates glycolysis through the up-regulation of key glycolytic genes, including *GLUT1, GLUT3, HK1, HK2,* and *LDHA* [148]. HIFs also reduce mitochondrial O_2_ consumption by attenuating glucose oxidation: they up-regulate the expression of pyruvate dehydrogenase kinase, which inhibits the activity of the pyruvate dehydrogenase complex (PDH complex), a key enzyme regulating the entry of pyruvate into the Krebs cycle through conversion to acetyl CoA [149,150]. Finally, the subunit composition of COX is altered in hypoxic cells by increased expression of the COX4–2 subunit, which optimizes the COX activity under hypoxic conditions, and increases degradation of the COX4–1 subunit by the LON protease, which optimizes the COX activity under aerobic conditions [146]. 

### 5.2. Metabolism of Endothelial Cells

The metabolism of ECs has been largely studied in the domain of cancer and atherosclerosis but is also of great interest in the problematic AVF. All ECs (arterial, venous, microvascular) are preferentially glycolytic, rather than OXPHOS since 85% of their ATP is produced by converting glucose into lactate [151,152]. One hypothesis is that downregulation of OXPHOS metabolism protects them from excessive ROS and oxidative stress [152]. At the same time, they save O_2_ to enhance the diffusion of oxygen to perivascular cells [152]. In addition, glycolysis produces less ATP but with faster kinetics, which is necessary for the rapid revascularization of hypoxic tissues when needed [153]. A hypoxic environment stimulates EC proliferation, and hypoxic proliferation of ECs increases glycolysis because of HIF-1 upregulation (both hypoxia- and growth factor- induced) [151].

### 5.3. Shear Stress, Hypoxia, and Metabolism in Endothelial Cells

Interestingly, hemodynamics and shear stress also play a role in EC metabolism. Laminar shear stress induces Kruppel-like factor-2 (KLF2) in ECs [154], whereas non-laminar (disturbed or turbulent) flow inhibits KLF2 (Figure 5). KLF2 is a zinc-finger transcription factor that maintains anti-proliferative and anti-inflammatory phenotypes of quiescent ECs. By inactivating p65 (subunit of NF-KB), KLF2 down-regulates cell adhesion (E-selectin) and prothrombotic molecules (thrombomodullin), inhibits VEGFR2, and up-regulates eNOS [155,156,157,158,159]. Doddaballapur et al. showed that laminar shear-stress induced KLF2 reduces glucose uptake and represses the key glycolytic enzyme 6-phosphofructo-2-kinase/fructose-2,6-bisphosphatase 3 (PFKFB3) [160]. This enzyme produces fructose-2,6-bisphosphate (F2,6P2), a strong allosteric activator of phosphofructokinase-1 (PFK1), a rate-limiting enzyme of glycolysis (Figure 5) [161]. Thereby, glycolytic metabolism is limited and blood flow keeps ECs in a resting state [162]. Kawanami et al. showed that KLF2 expression is also induced in hypoxic ECs, and over-expression of KLF2 inhibits HIF-1α (but not HIF-2α) stabilization and downstream expression of ANG2, IL-8 and VEGF [163]. Conversely, inhibition of KLF2 increases HIF-1α and its target-gene expression [163]. As such, KLF2 links hemodynamics, hypoxia and metabolism in ECs.

## 6. HIF and Vascular Diseases

In vascular pathology, hypoxia is typically the consequence of altered, destroyed or absent blood vessels leading to tissue ischemia. The mechanisms of the HIF response have therefore been studied in ischemic vascular diseases, such as myocardial infarction (consequence of obstruction of coronary arteries) or peripheral vascular disease (PAD, consequence of obstruction of arteries going to the limbs) [164,165].

Besides tissue ischemia, mechanisms of hypoxia and ischemia can occur within the vascular wall itself, with implications for diseases such as atherosclerosis [166], arterial aneurysm [167], chronic venous insufficiency [168] and thromboembolism [169,170]. There is growing evidence that the HIF pathway is implicated—for better or for worse—in the response of the vascular wall to inadequate oxygenation and/or increased cellular oxygen, a demand secondary to various stresses or injuries [171,172].

### 6.1. HIF and Atherosclerosis

Atherosclerosis is a condition in which fatty deposits (plaques) develop in the arterial wall, leading to stenosis and ultimately to thrombosis of the vessel [173]. Atherosclerotic plaques are mainly composed of cholesterol, other lipids, calcium, fibrin and inflammatory cells [174]. Oxygen diffusion through space-occupying atherosclerotic plaques could be limited due to wall thickening. Hypoxia could also originate from the high oxygen demand of metabolically active inflammatory cells, such as macrophage foam cells that play a pivotal role in this disease [175]. Detection of HIF-1α and activation of its target genes occurs in atherosclerotic plaques [166,176]. During atherogenesis (plaque build-up), hypoxia increases angiogenesis [166], releases pro-inflammatory mediators [177], activates MMPs [178], forms reactive oxygen species [179], and oxidizes low-density lipoprotein [180,181]. Necrotic cores within atherosclerotic plaques can lead to plaque instability and thromboembolic complications. The HIF-1α protein is found in tissue surrounding atherosclerotic arteries, inflammatory macrophages [166] and arterial SMCs around these necrotic cores [182,183]. Proangiogenic HIF-target genes, such as VEGF-A, are responsible for intra-plaque neoangiogenesis, contributing to plaque instability [184]. HIF-1 activity also alters the lipid metabolism of macrophages and vascular SMC [172,176,185]. Finally, besides hypoxia, several non-hypoxic stimuli of HIF have also been found in plaques and plaque macrophages, such as inflammation, ROS, vasoactive and thrombotic factors [129,186,187].

### 6.2. HIF and NH in Vascular Grafts

The role of the HIF pathway in the development of NH has also been studied. When atherosclerotic lesions occupy a long segment of the artery, surgeons frequently use a graft to bypass the lesion and provide blood to distal tissues. Either venous or prosthetic, these grafts are prone to NH. Although vein grafts and prosthetic grafts are not identical, because of different hemodynamics and oxygen tension, the characteristics of NH in vein grafts are similar to those of NH that form in AVFs [188]. In studies with animal models, grafts showed increased hypoxia within the vessel wall in regions of neointimal hyperplasia [189,190]. A study using a porcine model showed an association of HIF-1α expression with the degree of neointimal hyperplasia in grafts, and suggested that reducing hypoxia might inhibit venous NH formation, possibly by reducing the phenotypic switch of fibroblasts to myofibroblasts [191]. Paradoxically, increased activation of the HIF pathway may also protect against NH formation. Nakao et al. studied the role of carbon monoxide, a physiologically important vasodilator that acts via cyclic guanosine monophosphate, in the formation of NH [192]. They showed that a vein graft placed immediately after harvest led to less NH than if the graft was rinsed with lactated Ringer for 2 h prior to surgical placement. However, if the lactated Ringer solution was saturated with carbon monoxide, it significantly inhibited NH compared to the untreated control. The effects of carbon monoxide in inhibiting NH formation seemed to be associated with increased HIF-1α and VEGF expression a few hours after grafting. This effect, including the induction of VEGF, was reversed by treatment with YC-1, a HIF-1α inhibitor, supporting the conclusion that these effects were mediated through the activation of the HIF pathway. These apparently contradictory studies suggest that, at least in the setting of vein graft NH, transient activation of the HIF pathway may protect the wall from hypoxia, whereas chronic activation may maintain the ongoing NH process. Carbon monoxide may also display vasoconstrictor effects depending on oxygen and metabolic homeostasis, including ROS production, highlighting the important role of metabolism in the vascular wall [193].

### 6.3. HIF and NH in Vascular Access

Both prosthetic arteriovenous graft and AVF failure may be associated with increased activation of the HIF pathway. Misra et al. investigated HIF-1α and several target genes in stenotic segments from prosthetic grafts compared with AVFs [194]. The authors compared the expression of HIF-1α, several MMPs and TIMPs, and macrophage migration inhibitory factor (MIF), a proinflammatory factor that regulates proliferation and migration of vascular SMC, which is affected by HIF-1α in human vascular SMCs exposed to hypoxia [195]. Specimens from patients with prosthetic grafts expressed significantly more HIF-1α, MIF, TIMP-1, pro-MMP-2, and pro-MMP-9 compared with control veins. Pro-MMP-9 was also up-regulated in AVFs compared with control samples. The expression of MIF and TIMP-1 was significantly increased in prosthetic graft specimens compared with AVFs. Expression of HIF-1α in AVFs seemed to be higher than in controls although the differences reported in this study were not statistically significant. The authors concluded that there were major differences in the expression HIF-1α, and proteins regulated by HIF-1 in specimens removed from patients with prosthetic grafts and AVFs. They consequently developed porcine and mouse models of vascular access failure and found that HIF-1α and HIF-1-associated protein expression were increased in failed conduits compared with controls [196,197,198].

### 6.4. HIF Target Genes in Vascular Access

We previously studied oxidative stress and HIF-1 expression in a murine model of AVF maturation. Our team used a microarray analysis to show downregulation of 27 of the 83 genes involved in the OXPHOS pathway, downregulation of 12 of the 18 genes involved in mitochondrial long-chain fatty acid beta-oxidation, and 10 of the 13 genes involved in mitochondrial unsaturated fatty acid beta-oxidation, suggesting the presence of oxidative stress during early AVF maturation [199], and which was consistent with previous research [36]. We then showed increased excreted hydrogen peroxide, nitrotyrosine staining, and NOX-2 (**nicotinamide adenine dinucleotide phosphate** oxidase - 2) staining in the venous limb of the AVF during early maturation compared to control veins, confirming the presence of oxidative stress during AVF maturation. We also showed up-regulation of HIF-1α mRNA and protein expression in AVFs. Immunofluorescence of the AVF showed increased immunoreactivity of HIF-1-associated heme oxygenase-1 (HO-1) and VEGF-A, at both days 3 and 7 compared with control veins [199].

Interestingly, recent research has shown that expression of the HIF-1-target *HO1* is related to AVF function [200,201,202,203]. HO-1 is a cytoprotective and rate-limiting enzyme responsible for heme degradation, generating free iron, biliverdin, and carbon monoxide. Biliverdin is subsequently converted to bilirubin by biliverdin reductase, and free iron is rapidly sequestered by ferritin [204]. Bilirubin is a free radical scavenger that blocks lipid peroxidation [205]. HO has antioxidant, anti-inflammatory, anti-apoptotic, and angiogenic functions through its reactive products [98,204]. HO-1 is an inducible isoform, whereas HO-2 is expressed constitutively. Patients with *HO-1* gene polymorphisms characterized by long GT repeats, resulting in less HO-1 production, were more likely to have worse AVF patency [200,201]. In a murine model of AVF, Junco et al. showed that *HO-1* gene expression was markedly induced in the vascular SMCs. *HO-1* knockout mice had reduced patency and increased expression of pro-inflammatory and pro-oxidant mediators, such as MCP-1, MMP-2, and MMP-9 [206]. Subsequently, Kang et al., using the same model, showed that the adventitial delivery of an adeno-associated viral gene *HO-1* improved AVF blood flow and decreased venous wall thickness [203]. A functional AVF also requires HO-2 [202]. Shear stress also regulates HO-1 activity, with high flow inducing HO-1 to generate NO and mitochondria-derived hydrogen peroxide. Low flow induces lower levels of HO-1 that lead to macrophage infiltration and superoxide production within the vessel wall. However, the authors did not report whether these results were HIF-dependent. While they suggest an important role for HO in promoting outward remodeling and in preventing NH [207], we may think that HIF expression in this setting would be beneficial.

VEGF-A is necessary at low concentrations to promote endothelial cell health, NO and prostacyclin production, vasodilation, antithrombosis, and suppression of SMC proliferation but promotes angiogenesis and vasculogenesis at high concentrations [13]. Increased VEGF expression has been associated with early AVF thrombosis in human patients [208], and the VEGF-936C/C gene polymorphism has been associated with a 5.54-fold increase in risk of late AVF thrombosis [209]. Adventitial delivery of a lentivirus inhibiting VEGF-A expression decreases cell proliferation, inhibits NH and increases patency in a mouse model of AVF [34]. Systemic VEGF receptor gene transfer in rats decreases carotid artery restenosis, suggesting the utility of targeting this pathway to inhibit NH [210].

## 7. HIFs as Therapeutic Targets

Several experimental studies have shown that inhibiting the HIF-1 target gene VEGF decreases NH [34]. Further studies are needed to understand if the HIF pathway should be inhibited or activated in this setting. These studies can evaluate the broad range of therapeutic agents tested in other fields, in particular in cancer, since pharmacological targeting of HIF has been a major field of research these past two decades [101,211].

### 7.1. O_2_ Treatment

In a study using a rabbit iliac AVF model, Lata et al. demonstrated that NH is inhibited by the administration of 30% supplemental oxygen [212]. Based on the pattern of SMC proliferation, they suggested that 21 days of oxygen supplementation would be enough to control the initial burst of proliferation and subsequent NH. Further experiments by the same team demonstrated that HIF-1α was stabilized, VEGFR-2 up-regulated, and plasma VEGF-A was increased in rabbits with AVFs placed in normoxia [213]. In addition, these markers were significantly decreased when rabbits with AVFs were placed in 30% oxygen chambers for 21 days after surgery. Plasma collected in the hyperoxic group induced significantly less SMC and EC proliferation in vitro [213]. These results support the role of hypoxia and HIF-1 stabilization in NH. However, translation of this treatment to humans would mean wearing a nasal cannula all day. It is likely that it would be very difficult to convince patients who already carry a heavy medical burden to use supplemental oxygen therapy. Paradoxically, several studies conducted in different non-vascular experimental settings and cell types have shown that absolute hyperoxia induces HIF stabilization and activation of HIF targets [214,215,216,217,218]. The putative mechanisms may involve oxidative stress and ROS production, consequences of disturbed mitochondrial metabolism. In the AVF, both hypoxia (induced by the rupture of the vasa vasorum) and relative hyperoxia (induced by an increase in blood PaO2) may cause HIF stabilization, with a balance between the two mechanisms over the maturation period potentially leading towards NH and AVF dysfunction. Future studies may use ROS scavengers and/or regulate other metabolic targets to discriminate between the two mechanisms.

### 7.2. Pharmacological Treatment

Many existing drugs or drugs used in clinical trials can specifically, but mostly non-specifically, target HIF at different points of its regulation [219,220].

#### 7.2.1. HIF Inhibition

**Figure 6:**
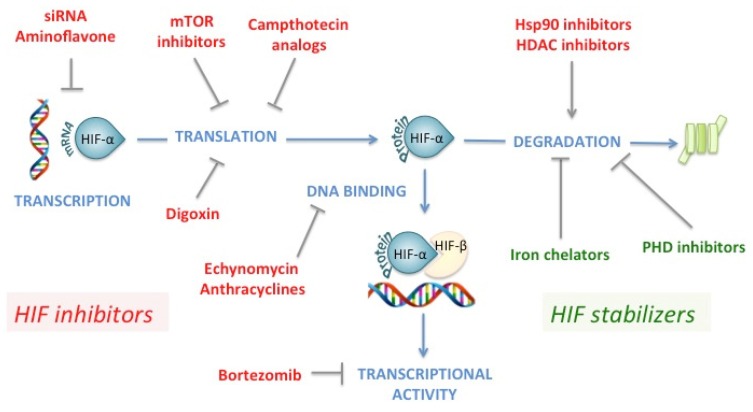
Pharmacological treatments targeting the HIF pathway. *Green: stabilization of HIF-α, red: destabilization of HIF*-α. *Blue arrows: physiological process, grey arrows: activation, grey T arrows: inhibition.*

According to their putative mechanism of action, HIF inhibitors can be divided into agents that modulate (Figure 6):

- HIF transcriptional activity, such as the FDA-approved chemotherapeutic drug PD-341 (Bortezomib, impairs binding of p300 to HIF1-α by enhancing the binding of FIH-1 to HIF1-α) [221],
- HIF-α mRNA expression, such as the experimental compounds EZN-2968 (antisense oligonucleotide) [222] and aminoflavone (ligand of aryl hydrocarbon receptor) [223],
- HIF-α protein translation, such as FDA-approved chemotherapeutic drug topotecan (campthotecin analog, inhibitor of topoisomerase I) [211,224], experimental compound EZN-2208 (derived from irinotecan, another campthotecin analog) [225], FDA-approved cardiac glycoside digoxin [226], and FDA-approved chemotherapeutic drugs temsirolimus and everolimus (mTOR inhibitors) [227,228,229],
- HIF-α protein degradation, such as Hsp90 inhibitors [230,231] or histone deacetylase (HDAC) inhibitors [232],
- HIF DNA binding, such as echinomycin (a cyclic peptide of the family of quinoxaline antibiotics) [233] and anthracyclines (such as the FDA-approved chemotherapeutic drug doxorubicin) [234].

#### 7.2.2. HIF Activation

One of the easiest ways to stabilize HIF-α is to act on the repressing enzymes PHDs and FIH-1. These enzymes being dependent on Fe(II) and 2-OG, iron chelators such as desferioxamine [34] and 2-OG analogs such as dimethyloxalylglycine (DMOG) [63] have been used in many experimental studies to stabilize HIF-1α. Importantly, these therapeutics are not isoform specific as the compounds inhibit all PHDs and therefore, all HIF-α. In the setting of atherosclerosis and treatment of PAD, experimental studies evaluated intramuscular injection of AdCA5 and intravenous administration of bone marrow-derived angiogenic cells that were cultured in the presence of the PHD inhibitor DMOG. The combined therapy increased perfusion, motor function and limb salvage in aged mice subjected to femoral artery ligation [165,235]. Based on these promising findings, a Phase I dose-escalation human clinical trial was performed using an adenoviral delivery system to deliver the active form of HIF1-α into the lower extremity of patients with critical limb ischemia. In addition, the therapy was well tolerated [236]. However, in a trial evaluating the efficacy of intramuscular administration of three different doses of Ad2/HIF1A/VP16 on walking time in patients with PAD and intermittent claudication, there was no significant clinical improvement when comparing the placebo and each HIF1-α dosage group [237]. This led to the conclusion that gene therapy with intramuscular administration of Ad2/HIF1A/VP16 was not an effective treatment for patients with intermittent claudication [237]. Although the reasons underlying failure remain unclear, concerns include the efficacy of gene transfer, including whether the gene transfer was increasing HIF to sufficient levels in the critical tissue compartment. For the treatment of CKD-induced anemia, promising compounds targeting PHD (Roxadustat, Vadadustat, Daprodustat, Molidustat) have been tested in patients with significant improvement of their hemoglobin level (mainly as a consequence of HIF-1-induced expression of EPO) [238,239,240]. These inhibitors are likely to become an important tool for the management of anemia in patients with CKD, where the oral route is a significant advantage. However, long-term safety of HIF activation must be determined, in particular with regards to HIF pathway tumorogenicity. Further studies are ongoing [238].

## 8. Conclusions

Increasing evidence shows that in AVFs, NH induced by upstream hemodynamic changes is linked to downstream metabolic disruption, involving the regulation of the response to hypoxia. Unfortunately, the common denominator of most HIF inhibitors and activators described so far is the lack of specificity, which indicates that they inhibit multiple targets and that HIF inhibition cannot be easily separated from other activities exerted by these agents [241]. Moreover, the majority of drugs primarily target HIF-1, although they may also act on HIF-2. One of the challenges of pharmacologically targeting the HIF pathway is to identify which isoforms are responsible for the disease and to find an isoform-specific drug. One of the most striking advances in the field is the successful targeting of HIF-2 in renal cell carcinoma, using a small molecule called PT2399, specifically designed to inhibit the binding of HIF-2α to the HIF-β subunit [242,243,244,245].

Besides finding HIF specific, isoform specific molecules, one major challenge will be to target delivery of these drugs to specific organs, to limit off-target and secondary effects.

## Abbreviations

ADM	Adrenomedullin
ADP	Adenosine DiPhosphate
AKT	Protein kinase B
AMF/GPI	Autocrine Motility Factor/Glucose-6-Phosphate Isomerase
AMP	Adenosine MonoPhosphate
ANG	ANGiopoietin
ARNT	Aryl hydrocrabon Receptor Nuclear Translocator
ATP	Adenosine TriPhosphate
AVF	ArterioVenous Fistula
bFGF	basic Fibroblast Growth Factor
BNIP3	BCL2/adenovirus E1B 19 kDa protein-Interacting Protein 3
CHIP	Carboxyl terminus of Hsp70-interacting protein
CKD	Chronic Kidney Disease
COX	Cytochrome C Oxidase
CREB	C-AMP Response Element-binding protein
DMOG	DiMethylOxalylGlycine
DNA	Deoxyribonucleic acid
EC	Endothelial Cells
ECM	Extra-Cellular Matrix
eNOS	endothelial Nitric Oxide Synthase
EPO	ErythroPOeitin
ESRD	End-Stage Renal Disease
ET-1	Endothelin-1
FAD	Flavin Adenine Dinucleotide
FDA	Food & Drug Administration
FIH	Factor Inhibiting HIF
GLUT	GLUcose Transporter
HAF	Hypoxia-Associated Factor
HDAC	Histone DeACetylase
HIF	Hypoxia-Inducible Factor
HK	HexoKinase
HO-1	Heme Oxygenase-1
HRE	Hypoxia-Responsive Element
Hsp	Heat-shock protein
ICAM	InterCellular Adhesion Molecule
IGF	Insulin-like Growth Factor
IL	InterLeukine
iNOS	inducible Nitric Oxide Synthase
KLF2	Kruppel-Like Factor 2
LDHA	Lactate DeHydrogenase A
LRP1	Low density lipoprotein recpetor-related protein 1
MCT	MonoCarboxylate Transporter
MMP	Matrix MetalloProteinase
mRNA	messenger RiboNucleic Acid
mTOR	mechanistic Target Of Rapamycin
mTORC	mTOR Complex
NAD	Nicotinamide Adenine Dinucleotide
NADH	Reduced form of NAD
NF-KB	Nuclear Factor-KB
NH	Neointimal Hyperplasia
NO	Nitric Oxide
NOX	**Nicotinamide adenine dinucleotide phosphate oxidase**
OPN	Osteopontin
PAD	Peripheral Arterial Disease
PDGF	Platelet Derived Growth Factor
PDH	Pyruvate DeHydrogenase
PFK	PhospoFructoKinase
PFKFB3	6-PhosphoFructo-2-Kinase/Fructose-2,6-Bisphosphatase 3
PHD	Prolyl Hydroxylase Domain
PI3K	Phosphatidylinositol-3-kinases
RACK	Receptor for Activated Protein Kinase C
ROS	Reactive Oxygen Species
SIRT	Sirtuin
SMC	Smooth Muscle Cells
TGF	Transforming-Growth Factor
TIE	Tyrosine kinase with Immunoglobulin-like and EGF-like domains
TIMP	Tissue Inhibitor of MetalloProteinase
TNF	Tumor Necrosis Factor
USRDS	United States Renal Data System
VCAM	Vascular Cell Adhesion Molecule
VEGF	Vascular Endothelial Growth Factor
VHL	von Hippel-Lindau
WSS	Wall Shear Stress
